# The prevalence and demographic associations of headache in the adult population of Peru: a national cross-sectional population-based study

**DOI:** 10.1186/s10194-024-01759-6

**Published:** 2024-04-03

**Authors:** Guiovanna Quispe, Cesar Loza, Luis Limaco, Ruth Gallegos, Carlos Palomino, Ivett Cruz, Jacqueline Miranda, Liliana Rodriguez, Andreas Husøy, Timothy J Steiner

**Affiliations:** 1 Neurology Service, Hospital Luis Negreiros Vega, Callao, Peru; 2 Department of Nephrology, Peruvian University Cayetano Heredia, Lima, Peru; 3 National Institute of Statistics and Informatics, Tacna, Peru; 4 Neurology Service, Hospital Daniel Alcides Carrion, Callao, Peru; 5 Neurology Service, Hospital Nacional Edgardo Rebagliati Martins, Lima, Peru; 6 NorHEAD, Department of Neuromedicine and Movement Science, Norwegian University of Science and Technology (NTNU), Edvard Griegs gate, Trondheim, Norway; 7 Department of Neurology, University of Copenhagen, Copenhagen, Denmark; 8 Division of Brain Sciences, Imperial College London, London, UK

**Keywords:** Epidemiology, Population-based study, Prevalence, Associations, Headache, Migraine, Tension-type Headache, Medication-overuse Headache, High altitude, Peru, South America, Global Campaign against Headache

## Abstract

**Background:**

The Global Campaign against Headache is conducting a series of population-based studies to fill the large geographical gaps in knowledge of headache prevalence and attributable burden. One major region not until now included is South America. Here we present a study from Peru, a country of 32.4 million inhabitants located at the west coast of South America, notable for its high Andes mountains.

**Methods:**

The study was conducted in accordance with the standardized methodology used by the Global Campaign. It was a cross-sectional survey using cluster randomised sampling in five regions to derive a nationally representative sample, visiting households unannounced, and interviewing one randomly selected adult member (aged 18–65 years) of each using the Headache-Attributed Restriction, Disability, Social Handicap and Impaired Participation (HARDSHIP) questionnaire translated into South American Spanish. The neutral screening question (“Have you had headache in the last year?”) was followed by diagnostic questions based on ICHD-3 and demographic enquiry.

**Results:**

The study included 2,149 participants from 2,385 eligible households (participating proportion 90.1%): 1,065 males and 1,084 females, mean age 42.0 ± 13.7 years. The observed 1-year prevalence of all headache was 64.6% [95% CI: 62.5–66.6], with age-, gender- and habitation-adjusted prevalences of 22.8% [21.0-24.6] for migraine (definite + probable), 38.9% [36.8–41.0] for tension-type headache (TTH: also definite + probable), 1.2% [0.8–1.8] for probable medication-overuse headache (pMOH) and 2.7% [2.1–3.5] for other headache on ≥ 15 days/month (H15+). One-day prevalence of headache (reported headache yesterday) was 12.1%. Migraine was almost twice as prevalent among females (28.2%) as males (16.4%; aOR = 2.1; *p* < 0.001), and strongly associated with living at very high altitude (aOR = 2.5 for > 3,500 *versus* < 350 m).

**Conclusion:**

The Global Campaign’s first population-based study in South America found headache disorders to be common in Peru, with prevalence estimates for both migraine and TTH substantially exceeding global estimates. H15 + was also common, but with fewer than one third of cases diagnosed as pMOH. The association between migraine and altitude was confirmed, and found to be strengthened at very high altitude. This association demands further study.

## Background

The Global Campaign against Headache, through a series of population-based studies, has over two decades endeavoured to fill the large geographical gaps in knowledge of headache prevalence and attributable burden [1]. One major region not until now included is South America. Here we present a study from Peru, conducted in accordance with the standardized methodology used by the Global Campaign [2].

Peru, with its 32.4 million inhabitants, is located at the west coast of South America [3]. It is highly urbanized: about four out of five of its population live in urban areas [3]. It is currently classified by the World Bank as upper-middle income [4], but there are inequalities and, at the time of this study, a poverty rate of 20% [5]. Rapid recent economic growth has substantially declined during the last decade [3], but benefited urban and coastal communities far more than rural communities in the Amazon and mountain regions (poverty rate > 55%) [3].

The prevalence and burden of headache in Peru have not been well described. In a search of the PubMed database for population-based studies, three were found [6–8], all conducted in selected high-altitude towns but with very dissimilar findings. The first found a migraine prevalence of 12.4% [6]; the second, surveying males only, found 32.2% had migraine and 15.2% tension-type headache (TTH) [7]; the third reported 1-year prevalences of 5.3% for migraine and 28.7% for headache [8]. In comparison, a recent meta-analysis including 32 population-based studies from Latin America and the Caribbean generated prevalence estimates for the region of 15% for migraine, 20.6% for TTH and 6% for “chronic headache”, with high between-study heterogeneity [9].

Our first aim, using established methods, was to estimate the 1-year prevalence of headache, overall and of the headache types of public-health importance (migraine, TTH and medication-overuse headache [MOH]), in the adult general population of Peru. Our second aim was to analyse associations with demographic variables. Important among these was altitude: the diverse topography of Peru, with settlements at both high and low altitudes, made it possible to revisit and possibly verify previous findings of a strong positive correlation between migraine and altitude [7, 8, 10].

## Methods

### Ethics

The study was conducted in accordance with the Declaration of Helsinki [11], with the protocol and study instruments approved by the Institutional Committee on Research Ethics of the Peruvian University Cayetano Heredia. All participants gave verbal consent to their inclusion in conformity with this approval.

Data were collected anonymously, and managed in compliance with data protection laws.

### Study design

This was a cross-sectional study of the general population of Peru aged 18–65 years. We followed the Global Campaign’s standardized methodology, which has been published in detail [2] and employed in many previous studies [1].

Accordingly, we used cluster-sampling to generate a sample representative of the population of Peru, with random selection of participants from communities in the five geographical regions of Cajamarca, Lima, Piura, Puno and San Martín. Trained health workers made unannounced door-to-door visits to randomly chosen households in each, during the period May to November 2019. From each household, the interviewers randomly selected and interviewed one adult member, following a structured questionnaire. Since only 22% of Peruvians lived outside urban areas [3], we deliberately oversampled rural areas to ensure sufficient statistical power for association analysis.

We aimed for a sample of *N* > 2,000 in accordance with guideline recommendations [2].

### Interviews and enquiry instrument

Interviews were performed using modules from the Headache-Attributed Restriction, Disability, Social Handicap and Impaired Participation (HARDSHIP) questionnaire developed by the Global Campaign [12], translated into South American Spanish in accordance with its translation protocol for hybrid documents [13].

The demographic module included age, gender, marital status, educational level, household income and employment. The headache modules began with neutral screening questions (“Have you ever had headache?” and “Have you had headache in the last year?”) and continued, when appropriate, with diagnostic questions based on ICHD-3 criteria [14] (directed towards the most bothersome headache when more than one type was reported). Specific questions on headache on the day preceding interview (“headache yesterday” [HY]) made it possible to estimate point (1-day) prevalence. Subsequent modules addressed attributable burden, but these are not reported here.

### Headache diagnoses

Diagnoses were derived algorithmically, following methods previously described [2, 12]. Participants reporting headache on ≥ 15 days/month (H15+) were first identified. Those also reporting a monthly consumption of acute medication on ≥ 15 days were diagnosed as probable MOH (pMOH); those not, were classified as “other H15+”, without attempt at further diagnosis. (The threshold for medication overuse was set at ≥ 15 days rather than ≥ 10 days [14] because the vast majority of the population in Peru would have access only to over-the-counter analgesics.) Remaining participants (with headache on < 15 days/month) were diagnosed, according to the algorithm, in the following order: definite migraine, definite TTH, probable migraine, probable TTH [14]. Definite and probable diagnoses were combined in adjusted prevalence estimates and association analyses.

### Analyses and statistics

Demographic characteristics of the sample were analysed descriptively and reported as proportions (%) with 95% confidence intervals (CIs) or using means and standard deviations (SDs). Gender was recorded as either male or female, age as a continuous variable, habitation as urban or rural. The male-female and urban-rural ratios were compared with those of the national population using chi-squared tests, and mean age using one-sample t-test.

Observed prevalences of headache overall, and of each type, were reported as proportions (%) with 95% CIs, along with age-, gender- and habitation-adjusted estimates taking account of distributions of these variables among Peru’s general population. Observed point prevalence was reported as a proportion (%). Predicted point prevalence was calculated from observed 1-year prevalence and mean headache frequency reported in days/month.

In association analyses for each headache type, caseness (positive for the type) was compared with non-caseness (all others in the sample). We used bivariate and multivariate analyses, calculating unadjusted odds ratios (ORs) and adjusted ORs (aORs), with 95% CIs, and included gender, age, habitation, altitude of dwelling, marital status, education level and household income. For these analyses, age was categorized into five groups (18–25, 26–35, 36–45, 46–55 or 56–65 years). Since two of the five sampled regions were at high altitude (Cajamarca: 2,750 m; Puno: 3,800 m) and three at or near sea level (Lima: 0 m; Piura: 55 m; San Martín: 340 m), we categorized altitude (≤ 350, 2,500–3000 and > 3,500 m). Marital status was categorized as single, married, or separated/divorced/widowed; education level as none, school, or college/university; and household income as < 600, 600–899, 900-1,299, 1,300-2,199 or ≥ 2,200 Peruvian nuevo sol (PEN) approximating to national quintiles (in June 2019, USD 1.00 = PEN 3.32).

We considered *p* < 0.05 as significant. SPSS version 28 was used for all analyses except for adjusted prevalence estimates, for which Microsoft Excel version 16 was used.

## Results

We visited a total of 2,385 eligible households, completing the survey in 2,149. The proportions from each of the five regions were: Cajamarca 339 (15.8%); Lima 647 (30.1%); Piura 396 (18.4%); Puno 447 (20.8%); San Martín 320 (14.9%).

The participating proportion was 90.1% overall, but varied by region from 82.1% in Lima to 96.5% in Puno.

### Descriptives

Gender distribution in the sample (1,065 males [49.6%], 1,084 females [50.4%]) matched that of the country’s population (chi-squared [1, *N* = 2,149] = 0.07; *p* = 0.80). Mean age in the sample (42.0 years ± SD 13.7) was somewhat higher than in the population aged 18–65 years (37.9 years [15]; t(df = 2148) = 13.9; *p* < 0.001). Because we deliberately oversampled rural participants (35.6%: see Methods), the urban-rural ratio in the sample differed from the national ratio (22% rural [15]; chi-squared [1, *N* = 2,149] = 231.1; *p* < 0.001).

### Prevalence

Reported lifetime prevalence (headache ever) was 83.6%, higher among females (87.5% [95% CI 85.3–89.4]) than males (79.6% [77.1–82.0]; *p* < 0.001). Observed 1-year prevalence of any headache was 64.6%, also higher among females (71.5%) than males (57.6%) (Table 1). TTH was the most common type (37.7%), with similar prevalence in each gender, while migraine, next most common, was almost twice as prevalent among females (28.2%) as among males (16.4%) (Table 1). H15 + was reported by 4.5%, females more than males, with about one quarter (1.1%) diagnosed as pMOH (Table 1).

In bivariate analyses we found associations of TTH and other H15 + with habitation (see below). Accordingly, we adjusted prevalence estimates with respect to population demographics to take account of habitation as well as age and gender. The effects were small: adjusted 1-year prevalence estimates were 65.5% for any headache, 22.8% for migraine, 38.9% for TTH, 1.2% for pMOH and a somewhat reduced 2.7% for H15+ (Table 1).

HY was reported by 12.1% of participants. In comparison, point prevalence of any headache predicted from the 1-year prevalence (64.6%) and reported mean headache frequency (4.2 days/month) was a rather lower 9.0%. Among those with headache, the proportion reporting HY was 18.7%: more than half (58.3%) of those with pMOH, almost half (48.6%) with other H15+, almost a quarter with migraine (23.7%), but fewer (11.6%) among those with TTH.

### Associations

The gender associations apparent in Table 1 are formally analysed in Tables 2 and 3, along with associations between the headache types and other demographic variables.

**Table 1:** Observed 1-year prevalences, and estimates adjusted for age, gender and habitation

Headache type	Observed			Adjusted for age, gender and habitation
	Overall	Male	Female	
	% [95% confidence interval]			
Any headache	64.6	57.6 [54.5–60.6]	71.5 [68.7–74.2]	65.5% [63.5–67.4]
Migraine	22.4 [20.6–24.2]	16.4 [14.3–18.8]	28.2 [2.6–3.1]	22.8% [21.0-24.6]
Definite	15.6 [14.1–17.2]	11.8 [10.0-13.9]	19.3 [17.0-21.8]	
Probable	6.8 [5.8–7.9]	4.6 [3.4-6.0]	8.9 [7.3–10.8]	
Tension-type headache	37.7 [35.7–39.8]	37.7 [34.7–40.6]	37.8 [43.9–40.8]	38.9% [36.8–41.0]
Definite	32.9 [31.0–35.0]	33.6 [30.8–36.5]	32.3 [29.5–35.2]	
Probable	4.8 [3.9–5.8]	4.0 [2.9–5.4]	5.5 [4.3–7.1]	
Probable medication-overuse headache	1.1 [0.7–1.7]	0.8 [0.4–1.6]	1.4 [0.8–2.3]	1.2% [0.8–1.8]
Other headache on ≥ 15 days/month	3.4 [2.6–4.2]	2.6 [1.8–3.8]	4.1 [3.0-5.4]	2.7% [2.1–3.5]

**Table 2:** Bivariate analysis of associations between headache types and demographic variables

Demographic variable	Migraine	TTH	pMOH	Other H15+
	Odds ratio [95% confidence interval]			
**Gender**				
Male (*n* = 1,065)	Reference	Reference	Reference	Reference
Female (*n* = 1,084)	**2.0 [1.6–2.5]** ***p*** ** < 0.001**	1.0 [0.8–1.2] *p* = 0.94	1.6 [0.7–3.8] *p* = 0.24	1.6 [1.0-2.5] *p* = 0.07
**Age** (years)				
18–25 (*n* = 325)	Reference	Reference	Reference	Reference
26–35 (*n* = 379)	1.1 [0.8–1.6] *p* = 0.50	1.2 [0.9–1.6] *p* = 0.26	1.3 [0.2–7.7] *p* = 0.78	2.0 [0.8–5.4] *p* = 0.15
36–45 (*n* = 558)	1.1 [0.8–1.5] *p* = 0.61	0.8 [0.6–1.1] *p* = 0.13	2.9 [0.6–13.5] *p* = 0.17	1.6 [0.6-4.0] *p* = 0.35
46–55 (*n* = 457)	0.8 [0.6–1.2] *p* = 0.28	1.2 [0.9–1.6] *p* = 0.18	1.4 [0.3–7.8] *p* = 0.68	1.4 [0.5–3.9] *p* = 0.48
56–65 (*n* = 430)	0.7 [0.5-1.0] *p* = 0.06	1.3 [1.0-1.7] *p* = 0.07	1.9 [0.4–9.9] *p* = 0.45	**3.1 [1.3–7.8]** ***p*** ** = 0.01**
**Habitation**				
Urban (*n* = 1,384)	Reference	Reference	Reference	Reference
Rural (*n* = 765)	1.1 [0.9–1.4] *p* = 0.40	**0.8 [0.6–0.9]** ***p*** ** = 0.006**	0.5 [0.2–1.3] *p* = 0.14	**2.0 [1.2–3.1]** ***p*** ** = 0.005**
**Altitude** (metres)				
ow < 350 (*n* = 1,363)	Reference	Reference	Reference	Reference
High 2,500-3,000 (*n* = 339)	1.2 [0.9–1.6] *p* = 0.24	**0.5 [0.4–0.6]** ***p*** ** < 0.001**	0.6 [0.2–2.1] *p* = 0.44	**2.5 [1.4–4.5]** ***p*** ** = 0.002**
Very high > 3,500 (*n* = 447)	**1.8 [1.4–2.3]** ***p*** ** < 0.001**	0.9 [0.7–1.1] *p* = 0.30	0.3 [0.1–1.4] *p* = 0.12	1.8 [1.0-3.2] *p* = 0.06
**Marital status**				
Single (*n* = 602)	Reference	Reference	Reference	Reference
Married (*n* = 1,252)	1.3 [1.0-1.6] *p* = 0.06	1.0 [0.8–1.3] *p* = 0.76	1.9 [0.6–5.8] *p* = 0.24	1.5 [0.8–2.7] *p* = 0.18
Widowed, separated, divorced (*n* = 295)	1.1 [0.8–1.6] *p* = 0.43	1.1 [0.8–1.4] *p* = 0.57	2.1 [0.5–8.3] *p* = 0.31	1.5 [0.7–3.3] *p* = 0.30
**Education level**				
none (*n* = 62)	1.1 [0.6-2.0] *p* = 0.70	0.7 [0.4–1.3], *p* = 0.27	2.8 [0.6–13.2] *p* = 0.21	2.6 [0.7–9.2] *p* = 0.15
School (*n* = 1,419)	0.9 [0.7–1.1] *p* = 0.30	0.9 [0.8–1.1] *p* = 0.34	0.8 [0.3-2.0] *p* = 0.66	**2.1 [1.1–3.8]** ***p*** ** = 0.02**
College, university (*n* = 668)	Reference	Reference	Reference	Reference
**Household income** (PEN)				
< 600 (*n* = 432)	1.0 [0.7–1.5] *p* = 0.84	**0.7 [0.5-1.0]** ***p*** ** = 0.04**	1.6 [0.2–15.1] *p* = 0.70	2.4 [0.9–6.3] *p* = 0.09
600–899 (*n* = 562)	1.1 [0.8–1.6] *p* = 0.61	0.9 [0.7–1.2] *p* = 0.51	1.2 [0.1–11.6] *p* = 0.87	1.1 [0.4–3.2] *p* = 0.82
900-1,299 (*n* = 518)	0.9 [0.6–1.4] *p* = 0.73	1.0 [0.7–1.3] *p* = 0.85	4.0 [0.5–31.4] *p* = 0.19	1.4 [0.5–3.9] *p* = 0.51
1,300-2,199 (*n* = 412)	1.1 [0.7–1.6] *p* = 0.66	1.2 [0.8–1.6] *p* = 0.41	4.4 [0.6–35.7] *p* = 0.16	1.7 [0.6–4.6] *p* = 0.33
≥ 2,200 (*n* = 225)	Reference	Reference	Reference	Reference

TTH: tension-type headache; pMOH: probable medication-overuse headache; H15+: headache on ≥ 15 days/month; significant associations are emboldened

**Table 3:** Multivariate analysis of associations between headache types and demographic variables

Demographic variable	Migraine	TTH	pMOH	Other H15+
	Adjusted odds ratio [95% confidence interval]			
**Gender**				
Male	Reference	Reference	Reference	Reference
Female	**2.1 [1.7–2.7]** ***p*** ** < 0.001**	1.0 [0.9–1.2] *p* = 0.73	1.6 [0.7–3.7] *p* = 0.30	1.7 [1.0-2.7] *p* = 0.05
**Age** (years)				
18–25	Reference	Reference	Reference	Reference
26–35	1.0 [0.7–1.4] *p* = 0.84	1.2 [0.9–1.7] *p* = 0.20	1.1 [0.2–7.1] *p* = 0.91	1.8 [0.7–5.1] *p* = 0.24
36–45	0.9 [0.6–1.3] *p* = 0.56	0.8 [0.6–1.1] *p* = 0.22	2.4 [0.5–12.6] *p* = 0.29	1.5 [0.5–4.1] *p* = 0.46
46–55	0.7 [0.5-1.0] *p* = 0.06	1.3 [0.9–1.7] *p* = 0.16	1.1 [0.2–6.7] *p* = 0.93	1.3 [0.4–3.7] *p* = 0.67
56–65	**0.6 [0.4–0.9]** ***p*** ** = 0.01**	1.4 [1.0–2.0] *p* = 0.05	1.3 [0.2–8.1] *p* = 0.77	**2.9 [1.0–8.0]** ***p*** ** = 0.04**
**Habitation**				
Urban	Reference	Reference	Reference	Reference
Rural	0.8 [0.6–1.1] *p* = 0.25	1.0 [0.8–1.3] *p* = 0.92	0.8 [0.2–3.2] *p* = 0.80	1.3 [0.6–2.6] *p* = 0.48
**Altitude** (metres)				
Low < 350	Reference	Reference	Reference	Reference
High 2,500-3,000	1.3 [0.9–1.9] *p* = 0.12	**0.4 [0.3–0.6]** ***p*** ** < 0.001**	0.7 [0.1–3.3] *p* = 0.64	1.6 [0.8–3.4] *p* = 0.20
Very high > 3,500	**2.5 [1.8–3.3]** ***p*** ** < 0.001**	0.8 [0.6-1.0] *p* = 0.08	0.4 [0.1–2.2] *p* = 0.31	1.3[0.7–2.8] *p* = 0.41
**Marital status**				
Single	Reference	Reference	Reference	Reference
Married	**1.5 [1.2-2.0]** ***p*** ** = 0.003**	1.0 [0.8–1.3] *p* = 0.89	1.7 [0.5–5.9] *p* = 0.37	1.0 [0.5-2.0] *p* = 0.95
Widowed, separated, divorced	1.3 [0.9–1.9] *p* = 0.19	1.1 [0.8–1.5] *p* = 0.60	1.8 [0.4–8.4] *p* = 0.45	0.9 [0.4–2.1] *p* = 0.74
**Education level**				
None	0.8 [0.4–1.5] *p* = 0.40	0.9 [0.5–1.7] *p* = 0.85	**7.1 [1.1–47.9]** ***p*** ** = 0.04**	1.1 [0.3–4.7] *p* = 0.87
School	**0.7 [0.5–0.9]** ***p*** ** = 0.01**	1.0 [0.8–1.5] *p* = 0.52	1.2 [0.5–3.1] *p* = 0.73	1.6 [0.8–3.3] *p* = 0.19
College, university	Reference	Reference	Reference	Reference
**Household income** (PEN)				
< 600	1.1 [0.7–1.8] *p* = 0.66	**0.6 [0.4-1.0]** ***p*** ** = 0.03**	1.1 [0.1–13.2] *p* = 0.95	1.2 [0.4–3.7] *p* = 0.77
600–899	1.1 [0.7–1.7] *p* = 0.60	0.9 [0.6–1.2] *p* = 0.43	1.0 [0.1–10.7] *p* = 0.99	0.6[0.2–1.9] *p* = 0.40
900-1,299	1.0 [0.7–1.5] *p* = 0.95	0.9 [0.6–1.3] *p* = 0.53	3.4 [0.4–28.2] *p* = 0.25	1.2 [0.4–3.4] *p* = 0.75
1,300-2,199	1.1 [0.7–1.7] *p* = 0.60	1.1 [0.8–1.5] *p* = 0.63	4.2 [0.5–34.0] *p* = 0.17	1.7 [0.6–4.8] *p* = 0.33
≥ 2,200	Reference	Reference	Reference	Reference

TTH: tension-type headache; pMOH: probable medication-overuse headache; H15+: headache on ≥ 15 days/month; significant associations are emboldened

In bivariate analyses, migraine was more common among females, as already noted (OR = 2.0; *p* < 0.001), but TTH showed no association with gender, and the other gender associations were not significant. Migraine became less prevalent after age 45 years, but this failed to reach significance even in the oldest group (OR = 0.7; *p* = 0.06). Other H15 + was more common in this group (OR = 3.1; *p* = 0.01), but no other associations between age and headache type were seen (Table 2). Again as noted, in these analyses there were associations with habitation: TTH less prevalent (OR = 0.8; *p* = 0.006) and other H15 + more prevalent (OR = 2.0; *p* = 0.005) among rural dwellers than urban. Marital status showed no associations; educational level appeared to be negatively associated with other H15+; income showed no clear associations (Table 2).

In the multivariate (adjusted) analyses (Table 3), female gender remained positively associated with migraine (aOR = 2.1; *p* < 0.001). The negative association of migraine with age became significant in those aged 56–65 years (aOR = 0.6, *p* = 0.01). The associations between habitation and TTH and other H15 + lost significance. Migraine was associated with being married rather than single (aOR = 1.5; *p* = 0.003). pMOH was most prevalent in those with no education (aOR = 7.1; *p* = 0.04), and (albeit not significantly, with small numbers) in those with mid-range income (Table 3). Those earning least had least TTH (aOR = 0.6, *p* = 0.03).

### Associations with altitude

In bivariate analyses, migraine was more prevalent at high altitude, and significantly so at very high altitude (> 3,500 m) (OR = 1.8; *p* < 0.001). Other H15 + was also more prevalent in the high-altitude areas. TTH on the other hand, was less prevalent at 2,500-3,000 m (OR = 0.5; *p* < 0.001) (Table 2). In multivariate analyses, the association between migraine and very high altitude was confirmed (aOR = 2.5; *p* < 0.001), while TTH remained less prevalent at high altitude (aOR = 0.4; *p* < 0.001).

## Discussion

This population-based study in Peru was the Global Campaign’s first such study in South America. It used the Campaign’s standardized methodology and questionnaire [2, 12], facilitating comparisons with studies elsewhere. We found, in summary, an age-, gender- and habitation-adjusted 1-year prevalence of headache of 65.5%, of migraine 22.8%, of TTH 38.9%, of pMOH 1.2% and of other H15 + 2.7%. Migraine was almost twice as prevalent among females (28.2%) as males (16.4%), and strongly associated with living at very high altitude (aOR = 2.5).

Over three quarters (78%) of Peruvians live in urban areas [3]. Those living in rural areas were therefore deliberately oversampled to include a statistically sufficient number. This was at the cost of representativeness within the sample of the country as a whole. Because, in bivariate association analyses, rural dwelling was negatively associated with TTH and positively with other H15+, we adjusted observed prevalences for habitation as well as for age and gender. These adjustments in fact made only small differences, indicating that our sample was, as far as could be judged, representative of the general population of Peru.

The findings indicate highly prevalent headache in Peru, as have those of similar Global Campaign studies elsewhere in the world [16–23]. The estimated prevalence of migraine (22.8%) in Peru considerably exceeds the current best estimate of global prevalence (14–15%) [24, 25]. The same is true of TTH (38.9% vs. 26.0% [25]). Relevant here is that this study, and all others referred to [16–23], took account, advisedly [2], of both definite and probable migraine and TTH, whereas many studies in the literature contributing to global estimates did not include probable diagnoses [24, 25].

Of those with H15+ (3.9%) in our study, fewer than a third (1.2%) were diagnosed as pMOH. This diagnosis was made entirely associatively (without evidence of causation, and hence only probable), based on reported headache and acute medication usage on ≥ 15 days/month, with reliance on the veracity of both reports. These are unavoidable limitations of cross-sectional studies. The estimate of 1.2% is within, and slightly towards the lower end, of the global range (1–2% [25–27]). However, the 3.9% with H15 + were not a low proportion, with all at risk of medication overuse. It is not evident that, in an upper-middle-income, highly urbanized country, there should be lack of access to acute medication as a bar to overuse. That this risk appeared unrealised in two thirds might be the result of public awareness and good health care, but also possible was under-reporting of medication overuse. Clinical studies are needed to ascertain the nature of the headaches in those with H15+: here we can only note that H15 + affects almost one person in every 25 of the adult population.

H15 + would have contributed substantially to the 12.1% observed 1-day prevalence of any headache (reported headache yesterday). This finding implied that almost one in eight of the adult population had headache on any particular day. It was, however, rather higher than the 9.0% point prevalence predicted from 1-year prevalence and mean frequency. The discrepancy suggests that participants underestimated headache frequency when recalling the number of attacks over the preceding 3 months. If this was so, they might well have underestimated medication use also.

As is almost invariably the case, migraine was strongly associated with female gender. Less usually, it was most common among those aged 18–35 years, with a consistent downward trend in prevalence thereafter, becoming least common – as is usual – among the oldest group (56–65 years). It was also most common in those with college or university education, for which there is no obvious explanation. Other H15+, into which any secondary headaches were likely to be captured, was most prevalent among the oldest group.

### Association of migraine with high altitude

Most interesting was the strong association of migraine with high altitude, confirming similar indications from earlier studies in Peru [7, 8] and a more definite finding in Nepal [10], a similarly mountainous country with settlements at both high and low altitudes. There were differences, however. In Nepal, sampling occurred over the range of altitude from near sea level to > 2,500 m, but only to a limited extent (*n* = 216) at higher altitude [16]. Here in Peru, we missed the middle range, but captured almost 800 participants dwelling at > 2,500 m. This was pertinent because, in Nepal, there was an approximately linear association between migraine prevalence and altitude up to 2,500 m; above this level there was evidence of decline, but higher altitudes were not investigated. Therefore, the finding here in Peru of a very strong association (aOR = 2.5) with very high altitude (> 3,500 m) is intriguing.

There is no clear explanation, but it presumably lies in the physiological compensations that occur at high altitude [16]. This demands further study.

### Strengths and limitations

This was a carefully conducted nationally representative study using standardized and well tested methodology in a large and adequate sample (*N* = 2,149) with a high participating proportion (90.1%). These were considerable strengths. The limitations were those always associated with cross-sectional questionnaire-based studies: dependence on recall, and limited ability to diagnose H15+.

## Conclusion

The Global Campaign’s first population-based study in South America found headache disorders to be common in Peru, with prevalence estimates for both migraine and TTH substantially exceeding global estimates. H15 + was also common, but with fewer than one third of cases diagnosed as pMOH. The association between migraine and altitude was confirmed, and found to be strengthened at very high altitude. This association demands further study.

## Data Availability

The original data are held on file at the Peruvian University Cayetano Heredia, Lima, Peru, and the analytical dataset at Norwegian University of Science and Technology. Once analysis and publications are completed, they will be freely available for non-commercial purposes to any person requesting access in accordance with the general policy of the Global Campaign against Headache.

## Abbreviations

aOR: adjusted odds ratio
CI: confidence interval
d/m: days/month
H15+: headache on ≥ 15 days/month
HARDSHIP: Headache-Attributed Restriction, Disability, Social Handicap and Impaired Participation questionnaire
HY: headache yesterday
ICHD: International Classification of Headache Disorders
MOH: medication-overuse headache
OR: odds ratio
PEN: Peruvian nuevo sol
pMOH: probable MOH
SD: standard deviation
TTH: tension-type headache
USD: United States dollar
